# Identification of a Polycystin-1 Cleavage Product, P100, That Regulates Store Operated Ca^2+^ Entry through Interactions with STIM1

**DOI:** 10.1371/journal.pone.0012305

**Published:** 2010-08-23

**Authors:** Owen M. Woodward, Yun Li, Shengqiang Yu, Patrick Greenwell, Claas Wodarczyk, Alessandra Boletta, William B. Guggino, Feng Qian

**Affiliations:** 1 Department of Physiology, Johns Hopkins University School of Medicine, Baltimore, Maryland, United States of America; 2 Department of Medicine, Division of Nephrology, Johns Hopkins School of Medicine, Baltimore, Maryland, United States of America; 3 Division of Genetics and Cell Biology, Dulbecco Telethon Institute, San Raffaele Scientific Institute, Milan, Italy; Cornell University, United States of America

## Abstract

Autosomal Dominant Polycystic Kidney Disease (ADPKD) is a genetic disorder resulting in large kidney cysts and eventual kidney failure. Mutations in either the PKD1 or PKD2/TRPP2 genes and their respective protein products, polycystin-1 (PC1) and polycystin-2 (PC2) result in ADPKD. PC2 is known to function as a non-selective cation channel, but PC1's function and the function of PC1 cleavage products are not well understood. Here we identify an endogenous PC1 cleavage product, P100, a 100 kDa fragment found in both wild type and epitope tagged PKD1 knock-in mice. Expression of full length human PC1 (FL PC1) and the resulting P100 and C-Terminal Fragment (CTF) cleavage products in both MDCK and CHO cells significantly reduces the store operated Ca^2+^ entry (SOCE) resulting from thapsigargin induced store depletion. Exploration into the roles of P100 and CTF in SOCE inhibition reveal that P100, when expressed in *Xenopus laevis* oocytes, directly inhibits the SOCE currents but CTF does not, nor does P100 when containing the disease causing R4227X mutation. Interestingly, we also found that in PC1 expressing MDCK cells, translocation of the ER Ca^2+^ sensor protein STIM1 to the cell periphery was significantly altered. In addition, P100 Co-immunoprecipitates with STIM1 but CTF does not. The expression of P100 in CHO cells recapitulates the STIM1 translocation inhibition seen with FL PC1. These data describe a novel polycystin-1 cleavage product, P100, which functions to reduce SOCE via direct inhibition of STIM1 translocation; a function with consequences for ADPKD.

## Introduction

Ca^2+^ homeostasis plays a vital role in the normal development of tubules in the mammalian nephron [1], [2]. Dysregulation of Ca^2+^ homeostasis is characteristic in the cyst formation associated with Autosomal Dominant Polycystic Kidney Disease (ADPKD)[3], but how dysregulation leads to cyst production is not well understood. ADPKD results from mutations in the polycystin genes PKD1 and PKD2/TRPP2 and mutations in their respective polycystin proteins, Polycystin 1 (PC1) and Polycystin 2 (PC2), both of which have been implicated as significant regulators of intracellular Ca^2+^ in renal tubules [4]. PC2 is a member of the transient receptor potential (TRP) family of ion channels [3] and has been demonstrated to be Ca^2+^ permeant in cilia, plasma, and ER membranes [3], [5], [6]. PC2 is known to regulate ER calcium permeability [7] and modulate IP3R [8] to lower ER Ca^2+^ stores. PC1's function in Ca^2+^ homeostasis is far less clear.

Tubular cysts result from a dysfunction in either PC1 or PC2, suggesting a common functional pathway. This idea is supported by evidence that PC1 must bind PC2 in order for PC2 to function as a Ca^2+^ channel [6]. A PC1, PC2 complex may function as a flow transducer on the primary cilium of epithelial cells [5], wherein flow transduction may be necessary for proper tubule alignment and formation [9]. However a second, less overt relationship may exist between PC1 and PC2, one revolving around a tight regulation of cytosolic and ER Ca^2+^, where a disruption of any Ca^2+^ regulator can lead to cyst formation. Normal PC2 expression and the resulting lower ER Ca^2+^ levels should increase Ca^2+^ influx and lead to apoptosis and increased cell proliferation [10], but does not. However, mutations or the loss of PC1 results in increased proliferation and apoptosis [2], suggesting that PC1 may be involved in regulating Ca^2+^ influx. PC1 may also play an important role in regulating cellular Ca^2+^ responses to stimuli, as PC1 has been reported to alter ATP activated intracellular Ca^2+^ increases by influencing the rate of Ca^2+^ reuptake in the ER [11].

ER Ca^2+^ store depletion is sensed by proteins of the stromal interaction molecule (STIM) family, specifically, STIM1 [12]. STIM1 is found predominantly in the ER membrane, where an EF hand region, located on its C-terminus within the ER lumen, binds ER Ca^2+^ molecules [12]. When ER stores are depleted, STIM1 re-localizes within the ER membrane to puncta opposite the plasma membrane [12], allowing for STIM1 to physically interact with Orai, a predicted subunit of store operated Ca^2+^ channels (SOC) [13]. STIM1, therefore, is thought to act as the ER Ca^2+^ sensor that directly activates the SOC channels to replenish ER Ca^2+^ levels.

Deciphering the role PC1 plays in Ca^2+^ homeostasis either at the ER or plasma membranes is further complicated by the cleavage of the PC1 protein and a lack of understanding about PC1 cleavage product localization. The first cleavage occurs constitutively and partially at the juxtamembrane GPS domain, generating a stable N-terminal fragment (NTF), and a C-terminal fragment (CTF) that coexist with uncleaved full-length PC1 [14]–[16]; the function of both products remains unclear. The second cleavage event previously described for PC1 results in a small C-terminal tail (CTT) fragment [17] that translocates into the nucleus, where it associates with β-catenin to disrupt canonical Wnt signaling [18]. PC1 localization is also controversial. Demonstrating PC1 activity on the cellular and cilia membranes has been the focus of the majority of PC1 work [1]–[3], however, there exists evidence that PC1 may also occur on the ER membrane [19], [20].

Here we describe a novel PC1 cleavage product, P100. We show that PC1, through its P100 product, inhibits Ca^2+^ influx at the plasma membrane, and we provide evidence that PC1 interacts with STIM1, preventing its re-localization after store depletion. Our data support the hypothesis that PC1 and P100 regulate store operated calcium entry by interfering with STIM1's ability to activate SOC channels on the plasma membrane.

## Results

### Analysis of polycystin-1 reveals novel endogenous cleavage product, P100

**Figure 1:**
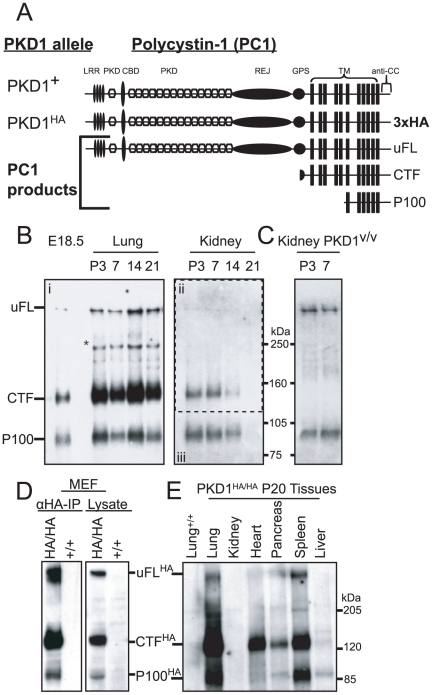
Biochemical characterization of a novel endogenous polycystin-1 product, P100. (A) Pkd1 alleles and schematic diagram of their corresponding polycystin-1 proteins. Pkd1^HA^ is a 3xHA-tagged Pkd1 knock-in allele that produces fully functional PC1 protein. The domains in polycystin-1 are shown. Anti-CC is directed to the cytoplasmic C-terminal tail. The position of uncleaved full-length (uFL), the C-terminal (CTF) and the P100 product is schematically shown. (B) Western blots for wild-type embryo (E18.5), lung (Bi), and kidney (Bii and iii) at different postnatal stages as indicated using anti-CC antibodies after immunoprecipitation. Western blot shown in Bii (enclosed by dashed box) originally from Yu et al 2007; © 2007 by The National Academy of Sciences of the USA. The bands corresponding to uFL, CTF and P100 are indicated. * indicates a band of unknown nature in the lung. (C) Western blot for postnatal kidneys from PKD1v/v mice. (D) Western blot from mouse embryonic fibroblasts (MEF) isolated from Pkd1^HA/HA^ and wild-type 11.5 day old embryos using anti-HA antibody after immunoprecipitation or on the whole cell lysate. (E) Western blot for homozygous Pkd1^HA/HA^ tissues at P20 using anti-HA antibody after immunoprecipitation. The lung of the wild-type littermate (+/+) serves as a negative control.

Using a PC1 C-terminal tail directed antibody (anti-CC-antibody, [16]) (Figure 1A), we detected by Western blot a novel PC1 product of approximately 100 kDa, here termed P100, in the embryo and postnatal mouse, in addition to the previously reported uncleaved full-length (uFL) and C-terminal fragment (CTF) (Figure 1Bi). We found that P100 is also expressed at various developmental stages of postnatal kidneys at levels commensurate to that of CTF: it deceased with the postnatal age and became undetectable at P21 (Figure 1Bii, iii). We detected P100 in the kidney of the Pkd1^v/v^ mice where PC1 cleavage at GPS is disrupted and CTF is thus absent (Figure 1C). We confirmed the authenticity of P100 with the HA-tagged PKD1 knock-in mice (Pkd1^HA/HA^), which express fully functional PC1 tagged with a 3xHA epitope at the C-terminus [21], and with 5XMyc tagged knock-in mice (Figure S1). We detected P100 in MEF derived from the Pkd1^HA/HA^ mouse by anti-HA after immunoprecipitation or directly in total lysate (Figure 1D). We found that P100 is expressed at different levels in the tissues of the developing mouse and its relative level to CTF also appears to differ among the tissues (Figure 1E).

**Figure 2:**
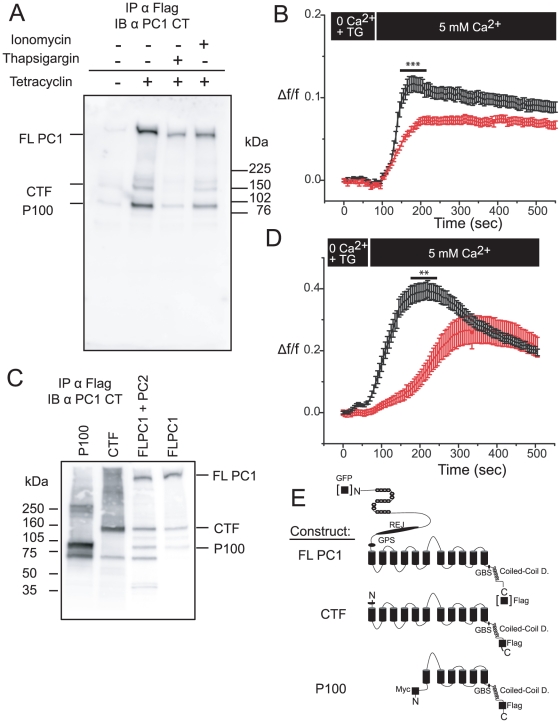
*In vitro* biochemical and functional characterization of polycystin-1 cleavage products. (A) Western blot of PC1-flag protein using anti-CT antibody after immunoprecipitation with flag conjugated beads from MDCK cells with stably transfected, tetracycline inducible PC1 expression. PC1 expression reveals three distinct bands: FL PC1, CTF, and P100. Right two lanes: the effect of 24 hours exposure of PC1 expressing cells to either 10 µM ionomycin or 40 nM thapsigargin (overnight). Blots representative of at least 3 experiments. (B) Fura 2-AM measurements of intracellular Ca^2+^ in either MDCK cells with (red; n = 5 coverslip, 99 cells) or without (black; n = 5 coverslips, 120 cells) tetracycline induced PC1-flag expression. Recordings began after cells reached steady state in a zero Ca^2+^, 4 µM thapsigargin ringers. The bath was then replaced with a 5 mM Ca^2+^ bath solution to measure the store operated calcium entry. Maximum SOC amplitudes (Δf/f) were compared (***p<0.001). (C) Western blot of PC1 and recombinant PC1 cleavage product constructs CTF and P100, in CHO cells. Protein was immunoprecipitated with flag conjugated beads then probed with anti-CT antibodies. Blot representative of at least three experiments. (D) Fura 2-AM measurements in CHO cells transiently transfected with GFP-PC1 (red; n = 12 coverslips, 65 cells) or with empty plasmid (black; n = 7 coverslips, 58 cells). Maximum SOC amplitudes (Δf/f) were compared (**p<0.01). (E) Cartoons of different PC1 constructs used in current study: Full length PC1 with either an N-terminus GFP or C-terminus flag tag; CTF construct with C-terminus flag tag; and P100 construct with both N-terminus myc tags and a C-terminus flag tag.

We detected similar PC1 products in a MDCK cell line with inducible Flag-tagged full-length PC1 expression under the control of a tetracycline sensitive promoter (Figure 2A) and in CHO cells (Figure 2C) after transient over-expression of full-length Flag-tagged PC1. This result indicates that P100 is likely generated by proteolytic cleavage at a site that is predicted within the third intracellular loop (Figure 1A) based on the apparent molecular size of P100 and therefore is the portion of PC1 that has sequence similarity to PC2 [22]. The relative amounts of the two cleavage products, CTF and P100 appeared to vary from blot to blot under seemingly similar experimental conditions. We found that 24 hour treatment of MDCK cells with 40nM thapsigargin appeared to reduced the amount of the CTF but not P100 (Figure 2A) whereas 10 µM of ionomycin did not; a result consistent with the hypothesis that ER Ca^2+^, not general intracellular Ca^2+^ levels, differentially affects the two cleavage processes.

Next we began an investigation into the possible functions of the PC1 cleavage products on Ca^2+^ homeostasis. It had previously been reported that PC1 inhibited ATP-induced capacitative calcium entry by increasing the rate of Ca^2+^ uptake to the ER, therefore reducing the stimulus for extracellular Ca^2+^ entry [11]. We found, however, that after depletion of the ER Ca^2+^ with thapsigargin in a zero Ca^2+^ bath, the reintroduction of Ca^2+^ in the control cells led to an increase in intracellular Ca^2+^ (Figure 2B) and this increase was significantly larger than in MDCK cells expressing PC1-flag (p<0.001). We confirmed the result in heterogeneous CHO cell populations after transient transfection with full length (FL) PC1-GFP (Figure 2D). Our results suggest that PC1 may also mediate inhibition of the extracellular Ca^2+^ entry when ER filling is prevented by thapsigargin and raises the possibility that PC1 may modulate SOCE current(s) directly at the cell surface. However, in both MDCK and CHO cells, PC1 was cleaved into CTF and P100. Could the cleavage products themselves be responsible for modulating the SOCE currents?

### CTF expression does not inhibit the SOC current

**Figure 3:**
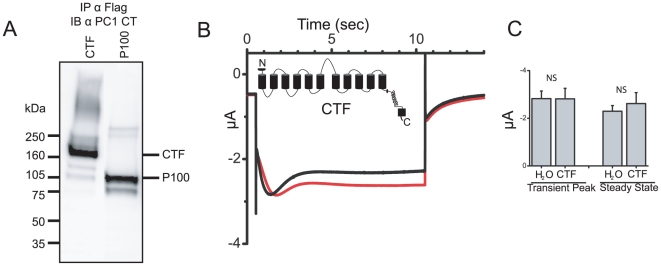
Polycystin-1 product CTF does not inhibit SOCE. (A) Western blot of PC1 cleavage product constructs P100 and CTF expressed in *Xenopus* oocytes, immunoprecipitated with flag conjugated beads and probed with anti-CT antibody. (B) Averaged currents elicited by a −120 mV voltage step in H2O injected control oocytes (black trace, n = 20) or from oocytes expressing CTF (red trace, n = 14). (C) Mean current amplitudes at the transient peak and steady state. ±SEM.

*Xenopus laevis* oocytes have well characterized Ca^2+^ activated Cl- currents (CaCC) [23], [24] and both voltage activated Ca^2+^ channels and store operated Ca^2+^ currents [24], [25]. We found that *Xenopus* oocytes, when subjected to a pretreatment with a zero Ca^2+^ solution containing thapsigargin, displayed large inward currents with complex kinetics (Figure S2). These “check” shaped currents were a combination of a fast transient, Niflumic Acid (NFA) sensitive, Ca^2+^ activated Cl- current; and a smaller but non deactivating, La3+ sensitive, store operated Ca^2+^ current (Figure S2). We used these endogenous currents of *Xenopus laevis* oocytes as an electrophysiological model to examine the role of the PC1 cleavage products in the regulation of SOCE currents. The *Xenopus* model system combines electrophysiological rigor with a cell type that does not express mammalian PC2, a protein shown to affect PC1 cleavage and expression of PC1 cleavage products [26], allowing a clear picture of any role PC1 cleavage products may have in regulating SOCE. We began with the larger CTF product (Figure 3A). Surprisingly, the expression of CTF in oocytes had no effect on either the transient peak (p<0.49) nor steady state currents (p<0.25) as compared to H2O injected controls (Figure 3B and 3C). To confirm that the lack of SOCE inhibition was not specific to *Xenopus* oocytes we expressed the CTF construct in CHO cells. In CHO cells first treated with zero Ca^2+^ ringers and thapsigargin, the reintroduction of extracellular Ca^2+^ resulted in comparable increases in Ca^2+^ influx in both the control and CTF expressing cells (Figure S3). These results suggest the CTF is not involved in inhibiting SOC entry. Interestingly, CTF is not further cleaved in *Xenopus* oocytes or CHO cells into a P100 sized product.

### P100 expression inhibits SOC currents

**Figure 4:**
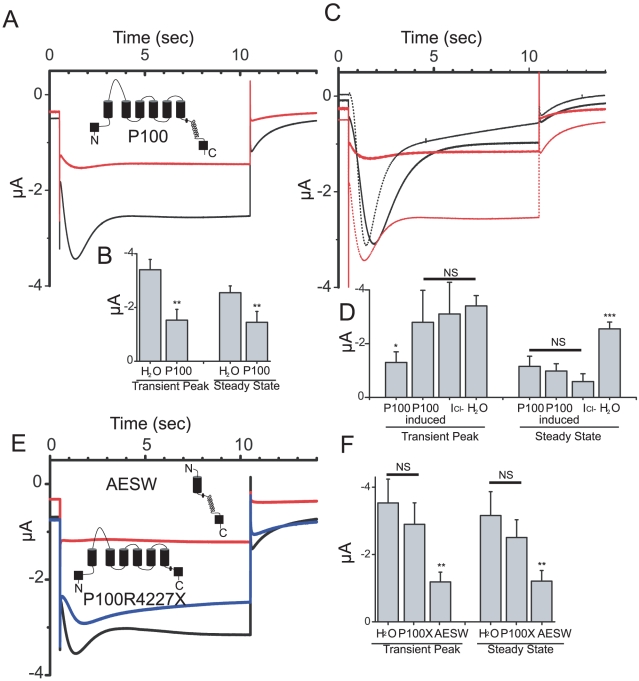
Polycystin-1 product P100 inhibits SOCE. (A) Averaged currents elicited by a −120 mV voltage step in H2O injected control oocytes (black trace, n = 38) or from oocytes expressing P100 (red trace, n = 18). (B) Mean current amplitudes at the transient peak and steady state. ±SEM; (**p<0.01). (C) Averaged currents form P100 injected oocytes (red trace, n = 11), and the same oocytes after three, 2 second, +60 mV pre-pulses (“induced” black trace, n = 11). For comparison the calculated NFA sensitive Cl- current from supplemental Figure S2E (black dotted trace) and the control currents from 4A (red dotted trace) are included. (D) Mean current amplitudes at the transient peak and steady state, including H2O control data from 4A and calculated I_Cl-_ from Figure S2E. ±SEM; (***p<0.001)(*p<0.05). (E) Averaged currents in H2O injected control oocytes (black trace, n = 38), oocytes expressing the AESW construct (red trace, n = 18), or oocytes expressing the P100 R4227X construct (blue trace, n = 13). (F) Mean current amplitudes at the transient peak and steady state; P100X is short for P100 R4227X. ±SEM; (**p<0.01).

Unlike CTF, the precise cleavage site for P100 is unknown so we created several constructs that start within the third intracellular loop. One of them, starting at V3645, produced a P100-like protein, which co-migrates with the P100 derived from the FL PC1 construct (Figure 2C and E) and can be considered a reasonable approximation of the P100. This construct was used for the subsequent studies of P100 function. In contrast to the larger CTF, P100 when expressed in *Xenopu*s oocytes, had a significant effect on the endogenous currents. In oocytes expressing P100, the check current was dramatically reduced as compared to H20 injected controls (Figure 4A). A comparison of current amplitudes at the transient peak and the steady state revealed P100 expressing cells showed a significant decrease in current amplitude (Figure 4B)(transient peak p<0.01; steady state p<0.01); suggesting both the CaCC current as well as the SOC current are being inhibited.

We next attempted to discern if P100 expression was affecting the SOCE only (secondarily affecting the CaCC), or affecting both the SOCE and the CaCC directly. *Xenopus* oocytes have well described voltage activated Ca^2+^ channels [23] which are responsible for the large outward CaCC currents seen when the cell is depolarized. The voltage gated Ca^2+^ channels were not activated during the negative pulse used to elicit the “check” currents, but were activated by positive pre-pulses, thereby providing a secondary source of Ca^2+^ for CaCC activation. Three, two second, +60 mV pre-pulses were delivered repeatedly to cells expressing P100, causing the induction of a check current (Figure 4C) significantly larger than the P100 suppressed current in the same oocytes prior to pre-pulse delivery. Interestingly, the induced current in the P100 oocytes was indistinguishable from the NFA sensitive Cl- current calculated in Figure S2. If compared to currents from H2O injected controls, the transient peak appeared similar, but the induced current dissipated, where as the control reached a steady state. An examination of the current amplitudes during the transient peak revealed the induced current was no different than the control peak (P<0.823)(Figure 4D). In contrast, the current amplitudes at the steady state revealed no induction or increase (p<0.15), but all three were significantly smaller than the control steady state current (p<0.001). These results strongly suggest that the CaCC currents are not being directly affected by the expression of P100, but are being inhibited indirectly, *via* an inhibition of the SOC current that is necessary for their activation at −120 mV.

To gain insight into whether the SOC current inhibitory effect of P100 may have physiological relevance, we used the mutant P100 expression construct bearing the disease-causing R4227X mutation and tested for the effect of the mutation on SOC current inhibition. The resulting mutant protein, P100 R4227X, lacks the C-terminal 76 amino acids containing the coiled-coil domain required for interacting with PC2 [6]. Expression of P100 R4227X in *Xenopus* oocytes did not significantly reduce the transient peak (p<0.50) or steady state currents (p<0.44)(Figure 4E and F). The importance of the missing tail region from the P100 R4227X mutant was confirmed by expression of an artificial PC1 product, AESW, containing only the final transmembrane domain and the C-terminal tail in *Xenopus* oocytes. AESW expressing oocytes displayed an inhibited transient peak (p<0.01) and steady state amplitudes (p<0.01) as compared to controls. Similarly, AESW transient over expression inhibited extracellular Ca^2+^ entry in thapsigargin treated CHO cells (Figure S3). These results indicate that the C-terminal sequence is required for the SOC inhibition and suggests that this activity of P100 plays a critical role for the physiological function of PC1.

### PC1 interferes with STIM1 puncta formation after ER Ca^2+^ depletion

**Figure 5:**
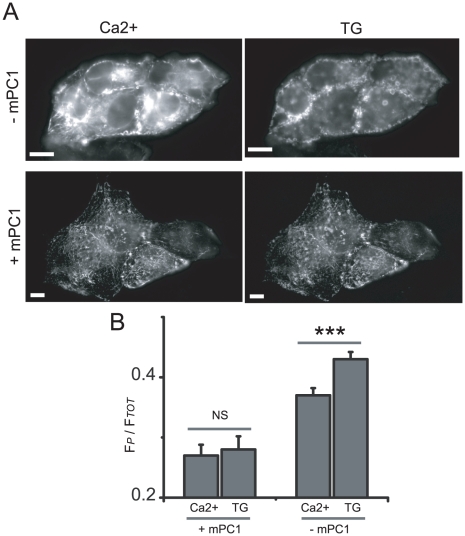
PC1 inhibits STIM1 translocation after ER Ca^2+^ depletion. (A) MDCK cells stably transfected with either mouse PC1 (+mPC1) or an empty vector (-mPC1) and transiently transfected with YFP-STIM1 imaged in 5 mM Ca^2+^ ringers and again after 15 min in zero Ca^2+^ with 4 µM thapsigargin. (B) Translocation of the YFP-STIM1 was monitored as the ratio of peripheral YFP signal (F_P_) to the total YFP signal per cell (F_Tot_). For mPC1 expressing MDCK cells, n = 4 coverslips, 12 cells; for control cells, n = 4 coverslips, 47 cells. Scale bar in images is 20 µM. ±SEM; (***p<0.001).

We hypothesized that PC1, through the actions of its P100 product, may exert its inhibitory effect on SOCE by interfering with STIM1 in the ER, retarding STIM1's ability to transduce a Ca2+ depletion signal to the SOCE channels of the plasma membrane. Functionally, we sought to address this by measuring the amount of STIM1 translocation to the periphery with or without the expression of PC1. We chose to use a MDCK cell line stably expressing mouse PC1, which we compared to empty vector control cells. The MDCK (with or without mPC1) cells were transfected with a YFP-STIM1 construct and photographed in high Ca^2+^ ringers (5 mM) after 15 minutes exposure to 4 µM thapsigargin (Figure 5A). Similar to a methodology used by Luik et al (2008)[27] the YFP signal of the periphery was compared to the total signal to create a F*_P_*/F*_Total_* ratio for the STIM1 fluorescence for before and after exposure to thapsigargin. In cells expressing mPC1, thapsigargin elicited no significant change in the STIM1 fluorescence ratio (p<0.298), suggesting that STIM1 did not translocate after ER store depletion. In the control cells with no mPC1 expression, thapsigargin induced a significant change in the STIM1 ratio (p<0.001). Interestingly, the expression of PC1 also limited the overall STIM1 fluorescence in the high Ca^2+^ ringer (p<0.001) in addition to the translocation of STIM1.

### P100 physically interacts with STIM1

**Figure 6:**
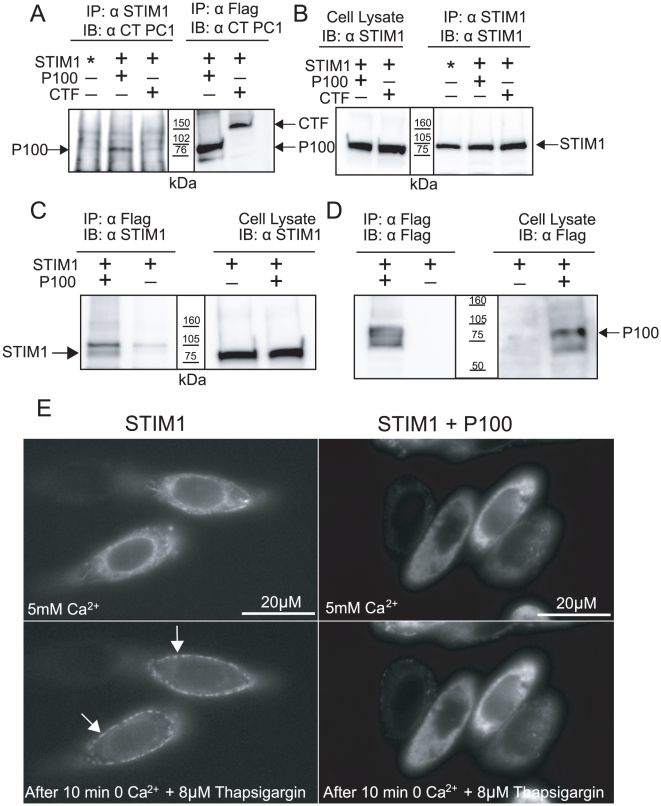
P100 physically interacts with STIM1. (A) CHO cells transiently transfected with STIM1 and either P100 or CTF construct; immunoprecipitated with anti-STIM1 antibody, then probed with anti-CT;or immunoprecipitated with flag conjugated beads then probed with the anti-CT antibody to verify transfection of PC1 products. * denotes endogenous STIM1 only. (B) STIM1 expression was verified by probing the same lysate and immunoprecipitate used in A and B with anti-STIM1 antibody. Blots representative of at least 3 experiments. (C) To confirm the pull down of P100 by STIM1, the reverse was attempted, pulling down STIM1 with P100. CHO cells were again transiently transfected with STIM1 and P100 or STIM1 alone. The Flag tagged P100 was immunoprecipitated with flag conjugated beads then probed with anti-STIM1 antibody. The expression of STIM1 was verified by probing the lysate directly with the anti-STIM1 antibody. (D) The expression of P100 was confirmed by probing the lysate and the immunoprecipitate with anti-Flag. Blots representative of at least 3 experiments. (E) CHO cells transfected with YFP-STIM1 and P100 (right) or the empty plasmid (left) and imaged first in a 5 mM Ca^2+^ bath solution (top) then again after a 10 min incubation in a zero Ca^2+^ bath with 8 µM thapsigargin (bottom). P100 retards the peripheral YFP puncta formation.

We over expressed STIM1 and P100 in CHO cells and found that P100 and STIM1 could be co-precipitated using an anti-STIM1 antibody, and visualized with the anti-PC1 CT antibody (Figure 6A). Consistent with the lack of SOCE inhibition, CTF was not pulled down with the anti-STIM1 antibody (Figure 6A). CTF and P100 expression was confirmed by immunoprecipitating the same lysate with Flag congregated beads then probing with the anti-PC1 CT antibody (Figure 6A). STIM1 expression was also verified under each condition (Figure 6B). The STIM1/P100 interaction was confirmed by reciprocal co-immunoprecipitation using flag-conjugated beads and visualized with the anti-STIM1 antibody (Figure 6C). The possible functional interaction of P100 and STIM1 was assessed like full length PC1 above. CHO cells were transfected with YFP-STIM1 and either an empty plasmid or P100 and photographed in 5mM Ca^2+^ ringers and again 10 minutes after exposure to 8 µM thapsigargin (Figure 6E). In CHO cells expressing YFP-Stim1 alone, ten minutes of thapsigargin treatment altered the STIM1 localization from a diffuse ER pattern to dramatic puncta around the periphery of the cell. However, the co-expression of YFP-Stim1 and P100 presents a different localization pattern, with very little of the puncta signal or YFP signal just beneath the plasma membrane. The inhibition of STIM1 re-localization in the presence of the P100 is not total, but does suggest that PC1 regulates Ca^2+^ entry through the generation of P100.

## Discussion

In this study, we have discovered a previously unrecognized endogenously expressed PC1 product of ∼ 100 kDa, P100, in various tissues including the kidney. Importantly, we also detected P100 in cells expressing recombinant PC1 with a full-length PKD1 cDNA expression construct. Together, these data indicate that P100 is most likely derived from proteolytic cleavage within the third intracellular loop and is expected to contain the final 6 transmembrane domains and the C-terminal tail, a segment of PC1 with significant sequence similarity to PC2. Interestingly, P100 is not produced in *Xenopus* oocytes or in CHO cells expressing only the CTF product, thus P100 cleavage may occur uniquely in the context of full-length PC1. One possible explanation for the differential cleavage of full length PC1 and CTF is that they are distributed in different subcellular locations with differing proteases. P100 is also present in Pkd1^V/V^ kidneys, indicating that P100 cleavage does not require GPS cleavage. Therefore, the full-length PC1 can act as a reservoir for generating CTF/NTF and P100 *in vivo*. PKD1^V/V^ mice have severely cystic kidneys at postnatal stages, but have no embryonic lethality in contrast to PKD1 knock-out mice [16]. The generation of P100 in Pkd1^V/V^ mice raises the possibility that P100 may help prevent various abnormalities during embryonic development such as cystic expansion of the kidney and pancreas observed in PKD1 knockouts, and therefore may have an important function during embryonic development.

Differential cleavage of full length PC1 and CTF could also be due to specific localization of a regulatory protein. Bertuccio et al (2009)[26] found that cleavage of PC1 into the CTT PC1 product is directly dependent on the expression of PC2. We also looked into the possible role of PC2 on P100 creation and found over expression of PC2 increased the amount of PC1 cleavage products, generally, but not any one specifically (Figure 2C). We also found, unlike Bertuccio et al (2009)[26], that ER Ca^2+^ levels had a small effect on the amount of PC1 cleavage, again generally, with no change in the CTF to P100 ratio. This could mean that ER Ca^2+^ levels or PC2 expression may not affect directly the P100 cleavage event, but more likely affect generally the stability of the PC1 products. The nature of the PC1 cleavage responsible for generating P100 is currently unknown.

We have also found that PC1 expression, and specifically P100, leads to an inhibition SOCE, an occurrence hereto unreported. PC1 mediated inhibition of SOCE could lower ER Ca^2+^ levels and shorten/reduce cytosolic Ca^2+^ transients [28], conditions known to increase resistance to apoptosis and proliferation [10]. Previously, SOCE inhibition has been directly linked to regulation of apoptosis and proliferation via multiple pathways [29]–[31]. In addition, many physiological ligands (*e.g.* ATP) act through the PLC/IP3 pathway to increase the release of Ca^2+^ form ER stores and raise cytosolic Ca^2+^. PC1, therefore, may attenuate the extent of net intracellular Ca^2+^ increases upon exposure to physiological stimuli. Li et al (2009)[20] showed that an ER localized PC1 fragment, QIF38 (identical to our P100), directly associates with IP3R to inhibit Ca^2+^ release from ER stores upon IP3 stimulation, an action that results in attenuated responses to exogenous ATP application in MDCK cells [20]. These findings taken with our own form a consistent picture of ER localized PC1 products acting to limit transient Ca^2+^ responses to physiological stimuli. Interestingly, a number of studies using the C-terminal tail of PC1 over expressed as a fusion protein have found the inverse; the expression of C-terminal tail leads to increases in intracellular Ca^2+^ and SOCE [32], [33]. However, in light of recent findings by Basavanna et al (2007)[34] who showed that the over expression of a PC1 C-terminus fusion protein acted as a dominant negative, these seemingly contrary findings may in fact be supportive of our hypothesis that PC1 expression leads to inhibition of the SOCE.

**Figure 7:**
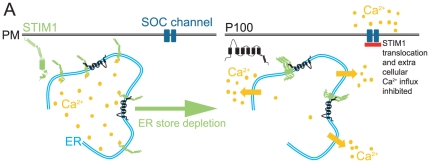
Model of P100 STIM1 interaction in the ER. (A) When ER Ca^2+^ stores are full STIM1 has Ca^2+^ bound and is evenly distributed throughout the endoplasmic reticulum (ER) membrane. Upon ER store depletion, STIM1 may oligomerize but does not translocate to areas of the ER near the plasma membrane (PM) when the polycystin-1 cleavage product P100 is present, inhibiting the activation of store operated Ca^2+^ (SOC) currents.

Can PC1's regulation of SOCE play a role in ADPKD? We found that a disease causing mutation in PC1, R4227X, does not inhibit SOC currents when expressed in *Xenopus* oocytes. These results suggest dysfunctional SOCE regulation (Figure 7) may disrupt normal Ca^2+^ homeostasis and therefore play a significant role in ADPKD. The same R4227X mutation also disrupts PC1/PC2 interactions [6] suggesting a role for PC2 in SOCE regulation.

Finally, we have demonstrated that expression of PC1 and P100 interferes with STIM1 translocation upon ER Ca^2+^ depletion. Normally, STIM1 in the absence of ER Ca^2+^ oligomerizes [35], [36] then secondarily translocates to form puncta at the ER-PM junction [35], [37], [38]. Oligomerization is dependent on the interactions of the EF-SAM regions located in the ER lumen portion of STIM1 [36], but translocation and puncta formation are dependent on the cytosolic polybasic [37], [38] and coil-coiled domains [35], [39]. Therefore, considering the C-terminal coil-coiled domain of P100, it seems most likely P100 interferes with the corresponding cytosolic domains of STIM1 which would predominately alter STIM1 translocation and puncta formation at the ER-PM junction (Figure 7). Further work on the involvement of the STIM1 coil-coiled domains in oligomerization would be necessary to confirm this prediction. Previously, the only reported physiological instances of either SOCE, CRAC, or specifically STIM1 suppression was during cell division, regulated by the phosphorylation of STIM1 [40]. Our data support an additional avenue of SOCE regulation, independent of cell division, a direct inhibition of STIM1 translocation by another protein, PC1. Although STIM1 has been shown to interact with a number of proteins (Orai and TRP channels), the PC1 interactions described here are among the first to describe a protein interaction that inhibits STIM1 function.

## Methods

Detailed methods can be found in the Supporting Information section (Methods S1).

### Electrophysiology

Whole cell voltage clamp recordings from the *Xenopus* oocytes were performed at room temperature in standard ND-96 ringers solution (in mM: 96 NaCl, 2 KCl, 1 MgCl_2_, 1.8 CaCl_2_, 5 hepes, pH 7.5). To increase the conductance of endogenous SOC currents, oocytes were pretreated with 4 µM thapsigargin (Sigma-Aldrich, USA) for up to 2 hours in the zero Ca^2+^ bath solution; generally, recordings were begun 3 minutes after the Ca^2+^ containing ND-96 bath solution was re-introduced to the oocytes. A holding potential of −40 mV was used on all oocytes.

### Mammalian Cells and culturing conditions

Stably transfected Madin-Darby canine kidney (MDCK) cells were a generous gift from Gregory G. Germino (NIDDK, Bethesda, MD USA) and cultured with selection agents hygromycin (100 µg/ml) and blasticidin (5 µg/ml).

### Plasmids and constructs

The human P100, the P100 R4227X, and the human PC1 fragment AESW constructs were also described previously under the names QIF38, R4227X, and AESW respectively [20]. The human STIM1 constructs, A151 and YFP-STIM1 were a generous gift from Guang Huang and Paul Worley (Johns Hopkins School of Medicine, Department of Neuroscience, Baltimore, MD USA).

### Fura 2 Ca^2+^ imaging and STIM1 images

CHO cells were rinsed with non-supplemented media then loaded with fura 2 (Invitrogen, USA). The cover slips were then bathed in a zero Ca^2+^ solution (in mM: 120 NaCl, 4.5 KCl, 1 EGTA, 2 MgCl_2_, 10 hepes, pH 7.4). Images were acquired once every 5 seconds using IPLab Software (BD Biosciences, USA). Thapsigargin (Sigma, USA, 4 µM) was used to deplete ER stores and high Ca^2+^ ringers (in mM: 120 NaCl, 4.5 KCl, 2 MgCl_2_, 10 hepes, and 3–5 CaCl_2_, pH 7.4) was secondarily applied to observe the store depletion activated Ca^2+^ entry. For STIM1 translocation assays, CHO cells expressing either YFP-STIM1 alone or with the CTF-100 construct began in high Ca^2+^ ringer then the bath was replaced with the zero Ca^2+^ ringer and 8 µM thapsigargin. For live images of STIM1 translocation in MDCK cells, MDCK cells stably transfected with either murine PC1 or the blank vector in addition to the YFP-STIM1 construct. 4 µM thapsigargin was used for store depletion. STIM1 translocation analysis was done as described by Luik et al (2008)[27]. All reported means are ± standard error of the mean (SEM).

## Supporting Information

Methods S1 Detailed methods.
(0.03 MB DOC)

Figure S1 Biochemical characterization of PC-1 product, P100 in Myc-tagged knock in mouse model. (A) PKD1 alleles and schematic diagram of their corresponding polycystin-1 proteins. PKD1^Myc^ and PKD1^ΔCMyc^ are 5xMyc-tagged Pkd1 knock-in alleles that produce fully functional PC1 protein. The domains in polycystin-1 are shown. The Pkd1^ΔCmyc^ allele produces 5xMyc-tagged truncated polycystin-1 protein with deletion of C-terminal 257 amino acids [21]. (B) Western blot for heterozygous Pkd1^myc/+^ and Pkd1^ΔCmyc/+^ and their wild-type littermate embryo (E17) using anti-Myc antibody after immunoprecipitation. The truncated CTF^ΔCmyc/+^ and P100^ΔCmyc/+^ are detected in the Pkd1^ΔCmyc/+^ embryo.
(1.73 MB EPS)

Figure S2 SOC and CaCC currents in *Xenopus laevis* oocytes. (A) Currents elicited from a representative H2O injected control *Xenopus laevis* oocytes 3–5 days after injection in a ND96 bath solution subjected to a +20mV step protocol from −100 mV to +60 mV from an initial holding potential of −40 mV. (B) Currents elicited from a representative H2O injected control oocyte pretreated with 4 µM thapsigargin in a zero Ca^2+^ ND96. (C) Summary steady state current/voltage relation from H2O injected control oocytes with (blue circles, n = 21) or without (red triangles, n = 13) thapsigargin/zero Ca^2+^ ND96 pretreatment. ±SEM; (**p<0.01). (D) Averaged currents in H2O inject control oocytes elicited by a 10 second voltage step to −120 mV from a −40 mV holding potential. For each cell (n = 4) currents were first recorded in zero Ca^2+^ ND96 (blue trace), then in the normal ND96 (1.8 mM Ca^2+^)(black trace) and finally in ND96 with 100 µM La^3+^ (red trace). Inset: mean current amplitudes at the transient peak (1.5 sec from beginning of record) and at the steady state (10 sec from beginning of record). ±SEM; (**p<0.01)(*p<0.05). (E) Averaged currents in H2O inject control oocytes elicited by a 10 second voltage step to −120 mV from a −40 mV holding potential. For each cell (n = 10) currents were recorded before (black trace) and after (red trace) replacing the bath with a low Cl- bath containing 100 µM Niflumic acid (NFA). Currents were recorded from a subset of cells (n = 4) further treated with 100 µM La^3+^ (blue trace). Subtraction of currents before and after treatment with NFA indicates the waveform of the NFA sensitive, probable Cl- currents (black dotted black trace). Inset: mean current amplitudes at the transient peak and steady state. ±SEM; (**p<0.01)(*p<0.05). (F) Measurements of transient peak current reversal potentials. Current/voltage relation of tail current amplitudes after initial −120 mV step in normal ND96 bath (black squares, n = 9), low Cl-/100 µM NFA bath (red circles, n = 9), or low Cl-/100 µM NFA/100 µM La^3+^ bath (blue triangles, n = 4). Inset: representative tail current measurements using reversal potential paradigm.
(3.58 MB EPS)

Figure S3 Expression of Polycystin-1 cleavage products have differing effects on store operated Ca^2+^ influx. Fura 2- AM measurements in CHO cells expressing PC1 cleavage products. Recordings began after cells reached steady state in a zero Ca^2+^, 4 µM thapsigargin ringers. The bath was then replaced with a high Ca^2+^ bath and Ca^2+^ influx through SOC channels is measured. (A) CHO cells transiently transfected with CTF (Red trace, n = 4 coverslips, 36 cells) or empty plasmid control (Black trace, n = 7 coverslips, 58 cells). (B) CHO cells transiently transfected with AESW (Red trace, n = 6 coverslips, 24 cells) or empty plasmid control (Black trace, n = 7 coverslips, 34 cells). ±SEM; (*p<0.05).
(0.82 MB EPS)
