# Supplementary material for: Identification of a Polycystin-1 Cleavage Product, P100, That Regulates Store Operated Ca2+ Entry through Interactions with STIM1
Source: PLoS One. 2010 Aug 23;5(8):e12305. doi: 10.1371/journal.pone.0012305 (PMC2925899; doi:10.1371/journal.pone.0012305)
Supplement: Methods S1 — Detailed methods. (0.03 MB DOC) [file pone.0012305.s001.doc]

**Methods S1. Detailed description of methods.**

***Xenopus laevis* oocytes**

Oocytes were removed from female pigmented *Xenopus laevis* (Xenopus 1 Inc.,

USA) and de-folliculated using collagenase A (Roche, USA) in a Ca2+-free OR-2 ringer solution. The following day, stage V-VI oocytes were selected and injected (Nanoinject II, Drummond Scientific, USA) with 50 nl of either mRNA or H2O (as a control). Oocytes after injection were cultured in a modified L-15 media (OR-3) and kept at 15-20°C. Experiments were done on the third, fourth, and fifth day after injection. mRNA was prepared using the SP6 mMessage mMachine (Ambion Inc., USA) according to the manufactures protocol. For RNA preparation and proper expression all cDNA constructs used in the oocytes were subcloned into the oocyte plasmid pCS2-MT.

**Electrophysiology**

Whole cell voltage clamp recordings from the *Xenopus* oocytes were made using the two electrode voltage clamp function of an Oocyte Clamp OC-725 amplifier (Warner Instruments, USA), performed at room temperature in standard ND-96 ringers solution (in mM: 96 NaCl, 2 KCl, 1 MgCl2, 1.8 CaCl2, 5 hepes, pH 7.5). Recordings were analog filtered at 500Hz (LHBF-48X NPI, USA), digitized at 5 kHz using a Digidata 1322A (Axon Instruments, USA), and analyzed using PClamp 9 software (Axon Instruments, USA). Electrodes are crafted from borosilicate glass and pulled to a resistance of 1 – 4 mega ohms and filled with a 3M KCl solution. For recordings, oocytes are immobilized on a plastic mesh within the recording chamber. Profusion was gravity fed, approximately 1 ml/min. Alternative bathing solutions were sometimes used, both a zero Ca2+ solution (in mM: 96 NaCl, 2 KCl, 1 MgCl2, 5 hepes, 1 EGTA, pH 7.5) and a low Cl- solution (in mM: 96 Na+ gluconate, 2 KCl, 1 MgCl2, 1.8 CaCl2, 5 hepes, pH 7.5). To increase the conductance of endogenous SOC currents, oocytes were pretreated with 4µM thapsigargin (Sigma-Aldrich, USA) for up to 2 hours in the zero Ca2+ bath solution; generally, recordings were begun 3 minutes after the Ca2+ containing ND-96 bath solution was re-introduced to the oocytes. A holding potential of -40mV was used on all oocytes. Niflumic Acid and Lanthanum were purchased from Sigma (USA).

**Mammalian Cells and culturing conditions**

Chinese Hamster Ovarian (CHO) cells were obtained from the ECACC and cultured in Ham’s F12 media (BioWhittaker/Lonza, USA) supplemented with 10% FBS and 1% penicillin/streptomycin, and split using 0.25% trypsin. Transient transfection of CHO cells was done using Lipofectamine 2000 (Invitrogen, USA) according to the manufactures protocol. Stably transfected Madin-Darby canine Kidney (MDCK) cells (a generous gift from Gregory G. Germino, NIDDK, Bethesda, MD USA) with tetracycline inducible PC1 or murine PC1 expression were cultured in DMEM with high glucose (GIBCO, USA) supplemented with 10% FBS and 1% penicillin/streptomycin, in addition to the selection agents, hygromycin (100 µg/ml) and blasticidin (5 µg/ml), and split using 0.25% trypsin. 2µg/ml tetracycline was applied for at least 24 hours for maximal PC1 / mPC1 induction.

**Plasmids and constructs**

The plasmids containing human FL PC1 (both N-terminal GFP and C-terminal Flag tagged) and the C-terminal fragment (CTF) with a C-terminal Flag tag were described previously [16]. The human P100, the P100 R4227X, and the human PC1 fragment AESW constructs were also described previously under the names QIF38 , R4227X, and AESW respectively [20]. The human STIM1 constructs, A151 and YFP-STIM1 were a generous gift from Guang Huang and Paul Worley (Johns Hopkins School of Medicine, Department of Neuroscience, Baltimore, MD USA).

**Biochemistry**

Western blots: In *Xenopus* oocytes, cells were first incubated for 30 minutes in lysis buffer (in mM: 20 Tris-HCl pH 7.5, 140 NaCl, 2% triton X-100, complete protease inhibitor) then homogenized using a hand held Pellet Pestle homogenizer (Kontes, USA). Lysates were then spun at 4500 g for 15 minutes and the supernatant recovered. CHO and MDCK cells were solubilized in lysis buffer (in mM: 20 Tris-HCl pH 7.5, 150 NaCl, 1% Nonidet P-40, 10% glycerol, complete protease inhibitor) spun at 12,000 rpm for 10 minutes and the supernatant retained. The protein concentration was established using the BCA protein assay kit (Thermo Scientific, USA). The solutions were separated on 4-15% or 3-8% SDS-PAGE gel and transferred to a PVDF membrane. Blots were probed with either anti-Stim1 / GOK (BD Biosciences, USA)(1:250), anti- PC1 CT [15](1:1000), anti-Flag (Sigma, USA)(1:500), or anti PC1-CC [16](1:1000). The blots were secondarily probed with HRP conjugated sheep anti (mouse or rabbit) IgG (1:3000). Occasionally Actin was used as a loading control (anti-actin antibody, 1:1000, Santa Cruz Biotechnology, USA). Blots were visualized using the Super-signal (Thermo Scientific, USA) chemiluminescence system.

Co-immunoprecipitation: CHO and MDCK cells were either stably or transiently transfected with various PC1 flag tagged constructs of interest, where in the Co-IP was performed to increase the specificity of a C-terminal antibody (anti-CT), allowing for the detection of all C-terminal cleavage products of PC1. Cells were solubilized in a Co-IP lysis buffer (in mM: 50 Tris-HCl pH 7.5, 150 NaCl, 1% Nonidet P-40, complete protease inhibitor), spun at 12,000 rpm for 10 minutes, and the supernatant collected. Anti-Flag conjugated M2-beads (Sigma, USA) were incubated with the lysate over night at 4°C with rotation. The following morning the beads were rinsed 5 times in 0.5 TBS then the Flag-Fusion protein is eluted in sample buffer, and separated on a SDS-PAGE gel, transferred, and probed with the anti-CT antibody as described above. In CHO cells a double transfection of various PC1 products and STIM1 was performed, and the lysates Co-IPed with the anti- Flag conjugated M2 beads as above or with the STIM1 antibody and protein A/G agarose conjugated beads (Santa Cruz Biotechnology, USA) , with the resulting blot probed with the Stim1 / GOK antibody or the anti-CT antibody.

**Fura 2 Ca2+ imaging and STIM1 images:**

CHO cells were gown on cover slips until 80 percent confluent, then transiently transfected with the construct of interest. For all constructs other than GFP-PC1, CHO cells were also transfected with a second GFP construct at one tenth the concentration to allow for visualizing cells that were successfully transfected. On the day of experimentation, the cells were rinsed with Ham’s F12 media (non-supplemented), then incubated in a solution containing 5µM of the cell-permeant acetoxymethyl (AM) ester of the calcium indicator fura 2 (Invitrogen, USA) for 30 minutes at room temperature. The fura 2 is made up in a stock solution of 50µg of fura 2 in a 10 µl solution of 1mg/ml pluronic-F/ DMSO. Following incubation, cells recovered for 30 min in minimum Ham’s F-12 media at room temperature. The cover slips were then inserted into the recording chamber, bathed in a zero Ca2+ solution (in mM: 120 NaCl, 4.5 KCl, 1 EGTA, 2 MgCl2, 10 hepes, pH 7.4) and mounted on the stage of an inverted Zeiss microscope (Zeiss Observer A1, Germany). A Zeiss FluorArc (Germany) was used to excite the cells at 340 and 380 nm, controlled via a Sutter (USA) Lamda 10-2 controller and filter wheel assembly, and the emission measured at 510 nm. All images were acquired using a Coolsnap CF CCD camera (Photometrics, USA) and fluorescence in each wavelength measured once every 5 seconds. Image acquisition, analysis, a wheel control were performed by IPLab Software (BD Biosciences, USA). Thapsigargin (Sigma, USA, 4µM) was used to deplete ER stores and a high Ca2+ ringers (in mM: 120 NaCl, 4.5 KCl, 2 MgCl2, 10 hepes, and 3-5 CaCl2, pH 7.4) was secondarily applied to observe the store depletion activated Ca2+ entry. Ca2+ imaging performed using MDCK cells was very similar to the protocol for CHO cells except that they needed 2 hours at 37°C for fura 2 loading and the media used was DMEM.

For live images of STIM1 translocation, CHO cells were transiently transfected with either YFP-STIM1 alone or with the CTF-100 construct (at 10X the concentration). Images were acquired using the same camera, microscope etc as the Ca2+ imaging described above. The cells began the experiment in the high Ca2+ ringer (5mM, see above) then the bath was replaced with the zero Ca2+ ringer (as described as above) and 8µM thapsigargin. Images were captured before thapsigargin addition, just after addition, 1 min after, 2 min after, 5 min after, and 10 min after thapsigargin addition.

For live images of STIM1 translocation in MDCK cells, MDCK cells stably transfected with either murine PC1 (expression under tetracycline control) or the blank vector were grown on glass cover slips until approximately 70-80 % confluent. They were then transiently transfected with the YFP-STIM1 construct. All imaging occurred at least 24 hours after STIM1 transfection and tetracycline treatment. The imaging methodology was similar to that used for CHO cells above except only 4µM thapsigargin was used, images were captured only before, at time zero, and after 15 min of treatment, and multiple cell clusters were imaged on each coverslip. STIM1 translocation analysis was done in manner similar to that described by Luik et al (2008)[27]. Briefly the YFP signal was measure for an entire cell before and after thapsigargin treatment, as well as the YFP signal just at the cell periphery. After background signal was subtracted from both measurements, a ratio of peripheral YFP signal to the total YFP signal was calculated using Photoshop (Adobe Systems Inc., USA) and the IPLab Software.

**Data Analysis:**

Electrophysiological recordings were analyzed using the Clampfit software (Axon Instruments, USA). CaCC amplitudes (transient peak) were measured 1.5 seconds after the beginning of a -120 mV pulse, the SOC current (steady state) amplitudes were measured 10 seconds after beginning of same pulse. Statistics used on the transient peak and steady state was a Student’s T test for pair wise comparisons of different cells, or a paired T–Test for comparisons of different treatments to the same cell, or an ANOVA, used with a Tukey’s Test for all multiple comparisons. Current reversal potentials were calculated by using a linear fit (OriginLab Corporation, USA) to the tail current amplitudes, where the zero current value corresponds to the reversal potential of the current. All reported means are ± standard error of the mean (SEM). Calcium imaging data was plotted also using OriginLab, and SOCE amplitudes were measured from their respective peaks.
